# Physical Activity as the Best Supportive Care in Cancer: The Clinician’s and the Researcher’s Perspectives

**DOI:** 10.3390/cancers14215402

**Published:** 2022-11-02

**Authors:** Cécile Torregrosa, Frédéric Chorin, Eva Ester Molina Beltran, Cindy Neuzillet, Victoire Cardot-Ruffino

**Affiliations:** 1 Oncologie Digestive, Département d’Oncologie Médicale Institut Curie, Université Versailles Saint-Quentin—Université Paris Saclay, 35, rue Dailly, 92210 Saint-Cloud, France; 2 Département de Chirurgie Digestive et Oncologique, Hôpital Universitaire Ambroise Paré, Assistance Publique-Hôpitaux de Paris, 9 avenue Charles de Gaulle, 92100 Boulogne Billancourt, France; 3 Laboratoire Motricité Humaine, Expertise, Sport, Santé (LAMHESS), HEALTHY Graduate School, Université Côte d’Azur, 06205 Nice, France; 4 Clinique Gériatrique du Cerveau et du Mouvement, Centre Hospitalier Universitaire de Nice, Université Côte d’Azur, 06205 Nice, France; 5 GERCOR, 151 rue du Faubourg Saint-Antoine, 75011 Paris, France; 6 Department of Cancer Immunology and Virology, Dana-Farber Cancer Institute, Boston, MA 02215, USA; 7 Department of Immunology, Harvard Medical School, Boston, MA 02215, USA

**Keywords:** physical activity, exercise, training, cancer, immunity, inflammation, metabolism

## Abstract

**Simple Summary:**

This literature review on adapted physical exercise has been written in order to raise awareness of medical staff to this central theme in the management of cancer patients, and very often left in the background after the specific treatment of cancer. We have summarized the benefits of exercise from a physiological, pathophysiological, and cellular point of view as well as through interactions between the person and their environment. In the second part, we explore the perspectives, based on a literature review.

**Abstract:**

Multidisciplinary supportive care, integrating the dimensions of exercise alongside oncological treatments, is now regarded as a new paradigm to improve patient survival and quality of life. Its impact is important on the factors that control tumor development, such as the immune system, inflammation, tissue perfusion, hypoxia, insulin resistance, metabolism, glucocorticoid levels, and cachexia. An increasing amount of research has been published in the last years on the effects of physical activity within the framework of oncology, marking the appearance of a new medical field, commonly known as “exercise oncology”. This emerging research field is trying to determine the biological mechanisms by which, aerobic exercise affects the incidence of cancer, the progression and/or the appearance of metastases. We propose an overview of the current state of the art physical exercise interventions in the management of cancer patients, including a pragmatic perspective with tips for routine practice. We then develop the emerging mechanistic views about physical exercise and their potential clinical applications. Moving toward a more personalized, integrated, patient-centered, and multidisciplinary management, by trying to understand the different interactions between the cancer and the host, as well as the impact of the disease and the treatments on the different organs, this seems to be the most promising method to improve the care of cancer patients.

## 1. Introduction

Today, the overall 5-year relative survival rate for all cancers combined, is estimated at 68% [1]. Progress in anticancer treatments yields an increase in patient survival, together with a better health-related quality of life (HRQoL), in most cases. However, the tumor mass and its systemic consequences are responsible for symptoms that can severely impair the HRQoL (e.g., malnutrition, pain, fatigue), along with the side effects of the medical and surgical treatments of cancer. These may persist for several years after the end of the treatments.

The accumulating evidence supports the benefits of physical exercise and nutritional management, during and after anticancer treatments, and these interventions are now recommended in routine practice [2,3]. Nutritional interventions are essential to prevent and reduce malnutrition, sarcopenia and cachexia [2,4,5]. Adapted physical activity is used in combination with nutritional interventions to fight malnutrition and to reduce the symptom burden related to the tumor and treatments. Multidisciplinary supportive care, integrating the dimensions of nutrition, exercise, and the psychosocial environment, alongside oncological treatments, is now regarded as a new paradigm to improve patient survival and the HRQoL [3]. Additionally, observational studies have shown that diet, physical activity, and weight control are associated with a decreased risk of cancer, and of recurrence and death after a cancer diagnosis, and preclinical models provide insight for specific antitumor effects of physical activity [6,7,8,9]. Recent guidelines have been published for nutrition and exercise (ESPEN, ASCO) and cachexia (ASCO, ESMO), in cancer patients. If the nutritional management is clear (well defined energy 25–30 kcal/kg/day) and protein (1–1.5 g/kg/day), the guidelines for APA (Table 1) are more general and less precise regarding what to propose to cancer patients in routine practice.

In this review, we propose an overview of the current state of the art physical exercise interventions in the management of cancer patients. We include a practical perspective with tips for routine practice. We then develop the emerging mechanistic views about physical exercise with a multiscale approach (at the host, microenvironment, and tumor cell levels), and their potential clinical applications.

Outline and Methods: In the first part of this review, we provide a summary of the current data from the guidelines, including the recent ones from ASCO and ESMO on cachexia, and an expert opinion on how to apply them in routine practice. In the second part, we explore the perspectives, based on a literature review. We performed a PubMed search with the following keywords: physical activity, exercise, training, cancer, immunity, inflammation, metabolism.

## 2. What Do We Know and Do in Routine Clinical Practice, in 2022?

### 2.1. Cancer Prevention

#### 2.1.1. Increasing Influence of Overweight and Obesity

Overweight and obesity promote the development of several types of cancer. The total annual number of cancer cases largely attributable to overweight or obesity is around 80,000 in the United States (nearly 6% of all cancers) and 60,000 in Europe [12]. Indeed, each 5 kg/m^2^ of the additional body mass index (BMI) above 25 kg/m^2^ is associated with an increase in the individual risk of cancer of the esophagus by 55%, of cancer of the endometrium by 52% and of kidney cancer by 31% [13]. To a lesser extent, the risks of cancer of the colon, rectum, pancreas and breast (in postmenopausal women) are also increased by being overweight. In addition, type 2 diabetes, which is often linked to overweight/obesity, is also associated with the risk of developing a broad spectrum of cancer types [14]. From a preventive point of view, weight loss (e.g., induced by bariatric surgery) is accompanied by a marked reduction in insulin resistance together with a decrease in the risk of overall mortality (−23%), with a drop in both deaths from cardiovascular diseases (−30%) and cancers (−23%) [15,16]. The effective management of overweight, particularly, visceral fat, is therefore an essential therapeutic means in the prevention of certain cancers [12]

**Figure 1:**
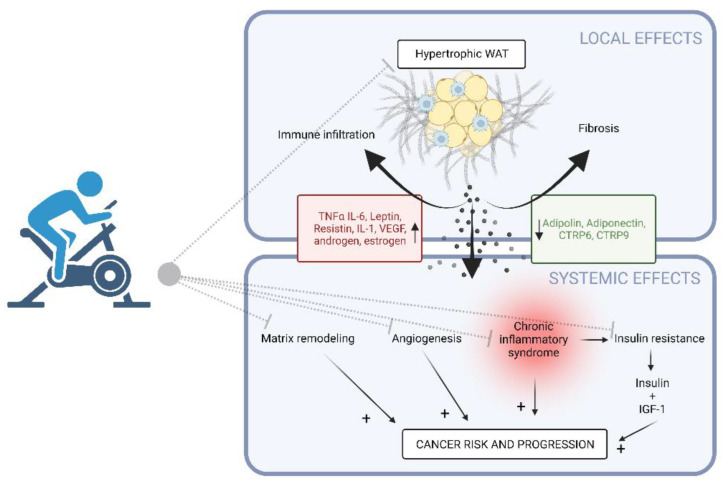
Local (immune infiltration, fibrosis) and systemic (matrix remodeling, angiogenesis, chronic inflammatory syndrome and insulin resistance) effects of hypertrophic white adipose tissue in obesity, on the cancer risk and progression and the potential inhibitory effects of exercise on these different changes. Black dots illustrate the gradient of adipokines and plus signs represent the different mechanisms contributing to cancer risk and progression. Created with BioRender.com.

The very abnormal physiology of visceral adipose tissue results in a strong secretion of pro-inflammatory cytokines and the deregulation of the secretion of some adipokines playing a major role in the onset of metabolic disorders (including insulin resistance) and the increased risk of cancer (Figure 1) [17]. A mild chronic inflammatory syndrome is common in obese people [18] and is linked to the hypersecretion of pro-inflammatory cytokines, including tumor necrosis factor alpha (TNF α) and interleukin 6 (IL-6) [19,20] by adipocytes and macrophages in the visceral fat. Moreover, this inflammatory syndrome, associated with an excess of free fatty acids, is responsible for insulin resistance and reactive insulin hypersecretion called hyperinsulinemia. Along with insulin, the secretion of the potent fraction of the free growth factor IGF-1 is increased and also acts as a pro-tumoral factor, inducing cell growth and reducing apoptosis. Leptin, an adipokine, and VEGF, one of the main pro-angiogenic factors, also secreted by adipose tissue, stimulate angiogenesis and the secretion of matrix remodeling enzymes called metalloproteinases, which further promote the cancerous invasion and metastatic processes. Tumor progression is even more promoted by the reduction in obese patients, of the concentration of another adipokine with antitumoral effects, adiponectin, which inhibits tumor cell growth, survival, and angiogenesis [21].

Finally, overweight/obesity is associated with an increase in the concentrations of androgens and estrogens [22], due to (i) lower concentrations of the transport proteins of these hormones leading to an increase in their free fraction, and (ii) the aromatization of androgens by the adipose tissue leading to estrogen production. These hormonal imbalances essentially explain the increased risk of developing hormone-dependent cancers, such as breast or endometrial cancer, in overweight and obese patients.

At the microenvironment level, the extracellular matrix (ECM) of the adipose tissue plays a role in maintaining tissue organization and as a reservoir for growth factors, cytokines and proteases, the local diffusion of which is modulated by ECM remodeling [23]. As obesity worsens and becomes chronic, adipose tissue undergoes profound remodeling, progressing to pathological alterations. In addition to a chronic low-grade inflammation, tissue fibrosis also sets in by the persistence of the activation of the myofibroblasts and of the fibrillar components originally produced to replace the normal tissue. Indeed, an accumulation of ECM, in particular fibrillar collagens, has been observed in the adipose tissue of obese persons [24]. Activation of TLR4 (toll-like receptor 4) initiated the signaling in macrophages present in the microenvironment of the adipose tissue, is involved not only in the maintenance of the local inflammation, but also in the development of fibrosis [25]. Adipose tissue dysfunction and fibrosis are possibly aggravated by local hypoxia [26]. These collagen deposits are organized around adipocytes (forming pericellular fibrosis), or in dense clusters, more or less thick, within the parenchyma [27] and they influence adipogenesis [28,29]. Adipose tissue has a critical role in energy homeostasis by meeting the body’s nutritional needs and adapting its perception so as to distribute energy to other organs, in the form of fatty acids (induction of lipolysis) or, on the contrary, by storing nutrients in the form of triglycerides. This functional plasticity of the adipose tissue turns out to be altered in fibrotic adipose tissue. The development of new tools measuring tissue elastometry, such as Adiposcan^®^ [30], has revealed that fibrotic changes in obese subjects were associated with changes in adipose tissue stiffness [31]. Moreover, Pellegrinelli et al. [32] demonstrated that fibrosis participated, through mechano-sensitive molecules, in the establishment of functional alterations of the adipocytes in obese patients (proinflammatory adipocytes, insulin resistance, disturbance of adipokine secretions). In addition, fibrosis in the adipose tissue seems to slow down weight loss after bariatric surgery in obese subjects [27]. Bel Lassen et al. [33] proposed a score for characterizing the extent of fibrosis at the peri-cellular level and around the adipocyte lobules from a subcutaneous adipose tissue biopsy (as a surrogate of visceral fat), allowing the quantification of fibrosis to be integrated into clinical practice. A high fibrosis score was associated with a poor weight-loss response after bariatric surgery, suggesting that adipose tissue fibrosis and dysfunction is a dynamic process that is not fully reversible when established. This kind of tool could make it possible to guide obese patients towards more personalized care.

Thus, obesity and overweight by increasing the proinflammatory cytokine release, promoting insulin resistance, altering the systemic metabolism, and inducing fibrosis, promote cancer initiation and development, making it a target of choice for the implementation of exercise in the cancer context.

#### 2.1.2. Direct Effect of Physical Exercise on Primary and Tertiary Cancer Prevention

The list of exercise-sensitive cancers, such as endometrial or colon cancers, is widely overlapping with cancers that are related to obesity or an unsuitable diet. This could be explained by the visceral fat mechanisms of action as described above (changes in insulin resistance, angiogenic and inflammatory factors, adipokines, and estrogens), amongst others. Indeed, exercise, by reducing visceral body fat, among other mechanisms, reduces chronic inflammatory syndrome, increases insulin sensitivity, especially in the muscles and liver [34], and ultimately reduces the risk of cancer. Moreover, other anti-tumor, visceral fat-independent, effects mediated, for example, by the immune system or the intestinal microbiota, have been described and will be developed later.

Concerning the effect of exercise on the cancer risk, two meta-analyzes have shown a significant reduction in the risk of cancer in people, with physical activity. The first on 12 prospective cohort studies, and including more than 180,000 cases of cancer, showed that the risk of 13 different cancers was lower in the 90th percentile of physical activity, compared to 10% of the population having less physical activity [10]. Similarly, the second meta-analysis, comprising 770,000 cases of cancer, revealed that physical activity reduced the risk of developing several types of cancer: colon, breast, endometrium, lung, esophagus and pancreas [35]. These observational studies, although they are adjusted on potentially confusing factors (e.g., BMI, tobacco smoking, alcohol…), are limited by their declarative nature, heterogeneity in the definitions of physical activity (e.g., leisure-time vs. total physical activity, intensity), and potential biases (i.e., people who are more active may have a healthier lifestyle). According to the 2018 WCRF/AICR report, the only cancers with a sufficient level of evidence for a protective role of physical activity are colon (relative risk [RR] 0.80–0.84), endometrium (RR 0.73) and postmenopausal breast cancers (RR 0.87). The WHO organization recommends engaging in at least 150 min/week of moderate intensity (i.e., a level that induces shortness of breath) physical activity. It is estimated in Europe that more than 1 in 2 adults and more than 2 out of 3 people over 65 years old are not active enough, according to current recommendations.

Epidemiology studies have also investigated the effect of physical activity intensity on the RR of cancer mortality. One study has shown that having a vigorous intensity physical activity (≥ 6 METs (Table 1)) regimen reduced the risk of cancer mortality by 64%, compared to a lack of physical activity. There is also a reduction in the risk of 44%, for moderate physical activity and 42%, for low physical activity [36]. The guidelines for the primary prevention also apply to the tertiary prevention setting. Being engaged in a healthy lifestyle, including regular exercise, is associated with improved survival and quality of life in cancer survivors [37] while a sedentary lifestyle (Table 1) is associated with an increased risk of mortality.

Despite the confusing factors that need to be adjusted in future studies, exercise seems to play a role in the prevention of different types of cancer.

### 2.2. Cancer Management and Treatments:

#### 2.2.1. The Threat of Malnutrition and Sarcopenia

The situation of patients during cancer treatment is totally different from the prevention setting. While overweight/obesity are a threat for cancer-free individuals, the major concern in patients with cancer is weight loss and malnutrition. Indeed, cancer is responsible for a dysregulation of the energy balance, which can lead to changes in body composition (loss of muscle mass and/or fat mass), and these have an impact on the HRQoL and patient survival. In fact, in cancer patients, an increase in resting energy expenditures and anabolic balance disturbance is frequently observed, due to cancer-induced hypercatabolism, inflammation, and insulin-resistance [38]. A decrease in energy intake is also frequent, as a consequence of the tumor syndrome (e.g., with dysphagia, malabsorption, pain, anorexia), or of the treatments (e.g., chemotherapy-induced digestive toxicity or sequelae of surgery). The increase in expenditures and the decrease in intake result in a negative energy balance, which, together with a decrease in physical activity, ultimately leads to malnutrition, sarcopenia and cachexia. Of note, a significant proportion of patients with cancer are obese, and they can be concomitantly malnourished or sarcopenic. The BMI is not reliable to screen malnutrition in these patients [4]. Assessment of muscle mass and function is particularly useful in this patient population.

Malnutrition is a frequent issue in oncology [39], affecting 39% of cancer patients and up to 60% of digestive oncology patients [40]. The Global Leadership Initiative on Malnutrition (GLIM) has developed a definition of malnutrition, based on objective criteria, in order to establish a consensus diagnosis, also recommended by the experts who established the ESPEN (European Society for Clinical Nutrition and Metabolism), ESMO (European Society for Medical Oncology) and ASCO (American Society of Clinical Oncology) guidelines. Malnutrition is defined by the association of one phenotypic criterion (reduced BMI, weight loss, or reduced muscle mass) and one etiologic criterion (reduced food intake/impaired nutrient uptake or inflammation) [41]. Malnutrition is often underestimated and underdiagnosed, leading to a delay in its management. It has multiple consequences, notably by altering the HRQoL of patients, reducing the tolerance and effectiveness of treatments, increasing the risk of complications, the number and duration of hospital stays, and the costs of care [42]. It is associated with a reduction in overall survival, representing a vital threat that should be considered by oncologists as important as the tumor burden itself. Martin et al. [43] showed, in a study including 8160 patients, that weight loss and the BMI independently predicted survival in patients with various cancer types (*p* < 0.01). Patients of stable weight with a BMI ≥ 25.0 kg/m^2^ had the longest survival [43]. Importantly, malnutrition is a dynamic process, which can be corrected by medical intervention at an early stage but becomes poorly reversible or refractory at a late stage. This justifies the early screening and management from diagnosis, as well as the regular reassessment as the disease progresses. Over the past two years, learned societies have given updated recommendations to increase oncologists’ awareness to screen and treat malnutrition [2,3,4,5,39,44].

Cachexia is defined as the disease resulting from malnutrition and interactions between the cancer and the host, involving chronic inflammation [45]. Cachexia includes objective components (e.g., weight loss, sedentary lifestyle, loss of muscle mass) and subjective components (e.g., anorexia, satiety, fatigue, etc.). Approximately half of all patients with advanced cancer experience cachexia [5]. Specific guidelines have been recently published by the ASCO ad ESMO. Sarcopenia is a direct result of cachexia. Cancer patients are at an increased risk of sarcopenia, which is defined by the European Working Group on Sarcopenia in Older People (EWGSOP), in 2010, as “a syndrome characterized by progressive and generalized loss of skeletal muscle mass and strength with risk of adverse effects, such as physical disability, poor quality of life and death”. The EWGSOP and TNCD (Thesaurus National de Cancérologie Digestive) recommends the documentation of low muscle mass associated with the criterion of low muscle strength or low physical performance, to establish the presence of sarcopenia [44,46]. Secondary sarcopenia is a prevalent condition in cancer patients, regardless of the disease stage [47] or the BMI [48], and is often associated with a higher mortality in both the late-stage [49,50] and early-stage of cancer [51]. Sarcopenia creates a vicious circle involving an increased risk of dose-related toxicities during treatment, resulting in a decreased treatment efficacy (due to the dose reduction or treatment discontinuation and a modified drug metabolism) and an increased risk of morbidity and mortality [52].

Comorbidities are also present in 30% to 80% of patients with cancer, such as type II diabetes or cardiovascular disease, and can contribute through a low-grade inflammation to an impaired metabolism and deconditioning [53].

Malnutrition and cachexia are really debilitating conditions that need to be evaluated and considered to better treat patients and propose adapted physical therapy.

#### 2.2.2. Pathophysiology of Cancer-Related Muscle Wasting and Dysfunction

The causes of cancer-related muscle dysfunction are complex, involving several factors related to the tumor, treatment, or lifestyle. Skeletal muscle plays a primary role in the disease prevention as an essential regulator of metabolic and inflammatory homeostasis [54], and muscle breakdown is a mechanism that appears early in cancer patients with repercussions throughout the body [55,56].

**Figure 2:**
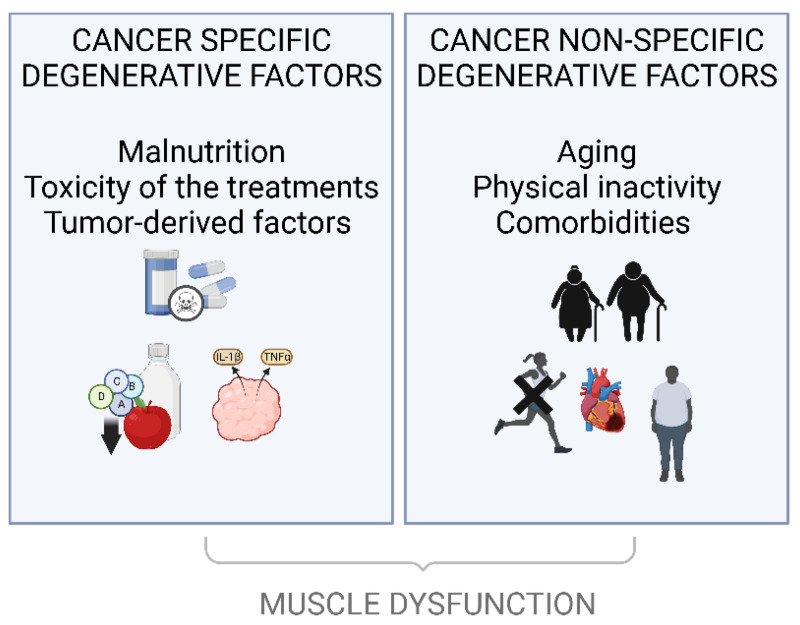
Cancer-specific (malnutrition, treatment associated toxicities and tumor derived factors) and non-specific (aging, physical inactivity and comorbidities) degenerative factors causing muscle dysfunction in patients with cancer. Created with BioRender.com.

Christensen et al. [57] showed that patients with cancer are subject to cancer-specific and non-specific degenerative factors, that are all causes of muscle dysfunction summarized in (Figure 2). Importantly, the age-related decline in muscle mass is observed as early as the end of the fifth decade and has been estimated at 1.9 kg per decade for men and 1.1 kg per decade for women [58]. In addition, the biological sex differences affect the microenvironment and intrinsic signaling of skeletal muscle (e.g., metabolism, mitochondrial function, immune response to injury, and regulation of myogenic stem cells). There is sexual dimorphism in the fiber type, function, in its regenerative capacity and estrogens have multiple molecular targets in muscle, in the context of cachexia (e.g., proteasome and mitochondria) [59]. A preclinical study established a link between the dysregulated ovarian function (acyclicity) and inflammation causing cachexia in an APC^min/+^ mouse model [60]. Androgens have been shown to have powerful anabolic effects on skeletal muscle. Preclinical work is still ongoing on the APC^min/+^ model [61], but observational and interventional studies strongly support an effect of androgens on the muscle mass in aging men, but the effects on muscle strength and especially physical function have been less clear [62,63]. This may explain the particularly high level of sarcopenia observed in prostate cancer patients (combination of age and hormonal deprivation). Moreover, 32% of cancer patients (at an advanced or localized disease stage) would be at “high nutritional risk” (score ≥ 3 on The Nutritional Risk Screening) [64] leading to an increased risk of muscle dysfunction. Other actors also play a significant role in muscle dysfunction, such as physical inactivity, but also factors that are directly linked to the pathophysiology of cancer and in particular linked to chronic inflammation [65] and toxicity of the treatments (e.g., reduction in appetite, disturbances in taste and smell, decreased upper gastrointestinal motility, nausea and constipation).

At the cellular and microenvironmental levels, cancer-related muscle dysfunctions include a decreased muscle fiber size predominating over type II [66], a mitochondrial dysfunction with a massive generation of ROS [67], as well as an inflammatory microenvironment. Mallard et al. [68] demonstrated, in 11 patients receiving chemotherapy for early-stage breast cancer, major mitochondrial alterations, including a reduced mitochondrial biogenesis, altered mitochondrial dynamics, potential defects in mitophagy, and an increase in the initiation of apoptosis. These alterations could partly explain the high prevalence of skeletal and cardiorespiratory muscle deconditioning classically observed in cancer patients.

Reports of the skeletal muscle status as a prognostic factor highlight the need for a better understanding of the complex etiology of muscle dysfunction in oncology.

Thus, muscle dysfunction is not only induced by cancer specific factors but also by patients’ specific degenerative conditions (aging, physical inactivity, comorbidities) that need to be taken into account.

#### 2.2.3. Roles of Physical Exercise during Cancer Treatments

Physical activity could help fighting against muscle wasting, cardiorespiratory deconditioning (which is the main cause of cancer-related fatigue) and some tumor- or treatment-related symptoms (such as pain, anxiety, nausea, sleep disturbance). The beneficial effects of physical exercise on depression, fatigue, anxiety, HRQoL and physical functioning have been largely demonstrated today. Moreover, more moderate evidence has been found for bone health and sleep quality [69]. Different other beneficial effects of exercise on the peripheral polyneuropathy induced by chemotherapy, cognitive function, falls, nausea, pain, sexual function and tolerance to treatment, have today low proof levels and require more research in these areas [69]. This evidence has been described in different types of cancer, during or after treatment (e.g., breast (majority), prostate, colorectal, gynecological, head and neck, lung or hematologic cancer) at an early stage or advanced disease [70,71,72,73,74,75,76,77,78,79,80,81,82,83].

The exercise intervention structured and supervised by a professional must be individualized for each patient, according to their preferences, disease, treatments and symptoms, to be feasible and safe for the patient, leading to the definition of “adapted physical activity” (APA). Two types of exercises are usually combined: endurance (repeated isotonic exercises that last in time to improve aerobic capacity (Table 1)) and resistance training (exercises training muscles against an external force, usually shorter than endurance training (Table 1)).

Endurance training has been shown to improve the cardiovascular function [84]. Indeed, aerobic exercise reduces inflammatory responses and ubiquitin-proteasome activity and permit to delay the development of heart failure-induced muscle atrophy [85]. Skeletal muscle mitochondrial activity is enhanced and increases the expression of PGC-1α, without a major impact on the skeletal muscle size [86]. Exercise activates the peroxisome proliferator-activated receptor (PPAR)-γ coactivator-1α (PGC-1α) pathway and facilitates the mitochondrial biogenesis. In addition, exercise reduces inflammation and prevents myocyte autophagy [87]. Plasma levels of free amino acids are reduced with exercise while proteins in fast-twitch skeletal muscle are increased. The mTOR/p70S6K pathway may be involved in this process [88].

Resistance training also targets the muscle fiber and its microenvironment. Following physical training, muscle mass and function in the elderly were significantly improved [89]. Therefore, the normalization of the muscle fiber and its microenvironment, through muscle strengthening and aerobic exercises, help to fight against sarcopenia and fatigue and to reverse the vicious circle of deconditioning.

Exercise emerges as a central tool in the supportive care to improve the HRQoL of cancer patients. Several prospective studies, but limited by their small sample size and high heterogeneity, have established a link between physical activity, nutrition and improved tolerance to treatment, a reduction of side effects and an improved quality of life in patients treated by chemotherapy [2]. Nevertheless, exercise could improve the HRQoL in patients with advanced cancer, by preventing the loss of function, helping to control symptoms, reducing symptoms, such as cancer-related fatigue, and maintaining daily autonomy. Moreover, recent controlled trials [70,71,72,73,74,75,76,77,78,79,80,81,82,83] showed that it is safe and feasible for this population of patients to have an adapted exercise intervention. (Table 2). Other studies are in progress, such as the study of Pudkasam et al. studying the physical, physiological and psychological impacts of self-directed physical activity, combined with self-monitoring and motivational interviewing on the quality-of-life patients who have had breast cancer, while being potentially inexpensive and widely applicable, if successful [90].

In the aftercare, the side effects of chemotherapy, such as fatigue, reduced muscular and cardiovascular performances, can last over time and prevent the resumption of professional activity. Exercise therapy is equally important for the person’s rehabilitation and reintegration into society, in particular by allowing a faster return to professional activities [91]. Observational epidemiological studies also show a decrease in the recurrence rate with a higher level of physical activity, compared to inactive people [92].

Overall, APA is expected to improve patient’s HRQoL and to help administer chemotherapy without reducing doses for toxicities [93], thereby possibly improving the treatment dose-intensity and patient survival. However, the scientific level of evidence is limited by the small sample sizes in most studies, and lack of standardization with heterogeneity of the proposed interventions. In addition, to be fully efficient, physical activity should be combined with nutritional intervention. Thus, it is necessary to determine a multimodal treatment plan for each patient to improve the HRQoL, survival and treatment tolerance in cancer patients. Future treatments could include pharmaceutical therapy, yet to date no validated drug is available, targeting the muscle breakdown and synthetic pathways, in addition to physical activity.

Physical activity integrated into patient care at present-day, aims at targeting cardiovascular and muscle functions, but also the quality of life and treatment toxicities.

### 2.3. In Clinical Practice, What to Do?

Physical activity and a balanced diet have become major tools for both the prevention and reduction of the risk of relapse, but also a tool for maintaining a correct HRQoL when the disease is metastatic. Physical inactivity should be prevented, and an active lifestyle should be promoted from the very beginning of the care pathway. It is recommended, in clinical practice, to set up appropriate physical activity in the primary prevention, during cancer treatment and in the aftercare, in order to reduce the risk of cancer, improve the treatment tolerance and decrease the risks of the disease’s recurrence. The specific exercise recommendations are described in the “Exercise Guidelines for Cancer Survivors”.

Indeed, programs must be progressive, regular and patient-specific. The proposed benchmarks are usually only medium to long-term objectives. This is why the notions of individualization and progressiveness are fundamental. The aim is to maintain the APA over the long term and to adjust the lifestyle behavior towards a “more active behavior”. It is recommended to practice progressively, both during a session and when resuming activity (by gradually increasing the amount of APA practiced). However, this notion of progressiveness is still too subjective (Table 3).

The American College of Sports Medicine (ACSM) Roundtable, published in 2010 and updated in 2019, reviews the exercise recommendations for cancer survivors [69,94]. The FITT criteria (frequency, intensity, duration and type) are developed to establish the exercise recommendations for aerobic and/or resistance training. *Exercise Guidelines for Cancer Survivors* described specific exercise recommendations [69]. The training program must be established for each person specifically, according to the type of cancer, the stage of the disease, the treatments, and the side effects. In addition to the general guidelines for physical activity, more specific exercises may be indicated for certain patients, in order to rehabilitate a particular function or physical condition (e.g., post-lung resection rehabilitation, muscular reinforcement during major deconditioning, disability secondary to a primary brain tumor, limb amputation, etc.). In this case, the dedicated programs will have precise objectives and will aim, in the long term, to enable the patient to regain a sufficient level of autonomy and then physical activity. If individual physical effort is a central component, supervision and implication of the sports coaches, are decisive for the success of an adapted physical activity for oncologic population. A review, published in 2017, reports that benefits on the HRQoL and body awareness are higher with a guided intervention for physical activity than with a self-guided activity [95]. In addition, the presence of a facilitator will make it possible to detect the appearance of signs of intolerance (muscular pains in the morning which persist throughout the day, significant and unusual fatigue, particularly in the morning, reduced alertness, and sleepiness during the day, etc.) which are warning signs of a poor tolerance to physical activity, and which require a temporary reduction in the program or in the activities spontaneously performed. It is necessary to invite previously highly active patients, with numerous sporting activities, to a certain moderation, in order not to expose them to the risk of the occurrence of signs of intolerance. In addition, during the session, the therapist provides concise instructions and ensures that the exercises are carried out in complete safety (segmental alignment, securing the environment, etc.) in order to avoid any joint constraints and risks of harmful effects. Each exercise begins with a phase of learning the movement to be able to carry out the exercise in safety, despite the addition of a notion of speed, explosiveness or loads. The control of breathing, which is part of the exercise, will be specified for each exercise, according to the modes of the muscular contraction (concentric, eccentric, etc.). The American Cancer Society’s *Nutrition and Physical Activity Guidelines for Cancer Survivors* recommends a healthy lifestyle that includes a healthy body weight, physical activity, and a balanced diet. Notably, after the diagnosis of stage III colon cancer, compliance with these recommendations was associated with longer survival [96].

Practical recommendations should be put in place to implement physical activity in the primary prevention and tertiary prevention (after cancer). In the primary prevention, the current recommendations are to complete 150 min per week of moderate intensity training, combining aerobic and resistance exercises [96]. One of the rules of effective physical activity programs is regularity. It is better to do 30 min on four different days than 120 min on one day, followed by several days without physical activity. It seems obvious that it is better to do it as regularly as possible than to make up for lost days in one go. A particular situation for which the recommendations are precise, is the perioperative situation, and in particular, within the framework of gastric and pulmonary cancers. A physical activity program is implemented during the neoadjuvant chemotherapy, to optimize the patient’s functional abilities so that they arrive at surgery in the best possible physical condition with maintenance of the cardiorespiratory capacity (which is known to be correlated with the risk of complications) and to avoid postoperative physical deconditioning, in order to resume the therapeutic sequence (adjuvant chemotherapy) in the best possible conditions [2]. During the treatment of cancer, the recommendations are less standardized and depend mainly on the general condition of the patient. In the advanced stages of the disease, physical activity is a central supportive care tool aimed at improving the quality of life, but also reducing symptoms and improving tolerance to treatment [92,97].

It is important to combine physical activity with nutritional interventions for optimal effect, particularly in malnourished patients, regardless of the stage of the disease [2]. Particularly in a perioperative situation, where undernutrition is frequent (may affect up to 50% of patients) and is responsible for an increase in morbidity, in particular infectious and postoperative complications, mortality and length of hospitalization. The recommended perioperative intakes are 25 to 30 kcal/kg per day including 1.2 to 1.5 g of protein per kg with a carbohydrate-lipid caloric ratio of approximately 60/40%, whatever the primary cancer. In malnourished patients, nutritional support is essential before surgery, even if it should not delay it. An evaluation by a nutritionist dietician with the implementation of nutritional support after possible correction of hydro-electrolyte disorders is recommended.

### 2.4. Unmet Needs

Despite numerous studies focusing on “physical activity in oncology” and nutrition, only one in two patients today participated in sports and exercise therapy, during medical treatment, or were not informed of sports and exercise therapy options. One of the reasons is related to the lack of commitment to APA by people with cancer. This can be explained by different types of barriers: physical (fatigue, pain), environmental and organizational (time, geographical, financial constraints), and psychological (negative beliefs, lack of motivation). However, the modification of these barriers is not systematically accompanied by a change in behavior. A meta-analysis attempted to determine which strategy was increasing breast cancer survivor motivation to take up physical activity. Ideal for increasing participants’ motivation, seems to be a combination of a step tracking tool, combined with motivational interviewing [98]. Generally speaking, the interest in developing the feeling of self-efficacy (confidence in one’s physical abilities) and the level of self-determination of individuals (feeling at the origin of one’s behavior), notably through the use of the motivational interview techniques, and in changing their beliefs about the effects of physical activity (balance of risks and perceived benefits) appear to promote commitment. Nevertheless, these interviews are not systematic.

Finally, the results of the studies suggest the implementation of early information on physical activity, disseminated at the beginning of the treatment process, in order to make patients aware of the possibility of initiating physical activity in a safe manner and of the expected beneficial effects. Practice during the treatment period seems nevertheless more complex to envisage, especially when patients did not practice APA regularly before the disease. Thus, the interventions proposed during this period will have to take into consideration, in addition to the specific effects of the treatments, the individual reticence and barriers.

**Figure 3:**
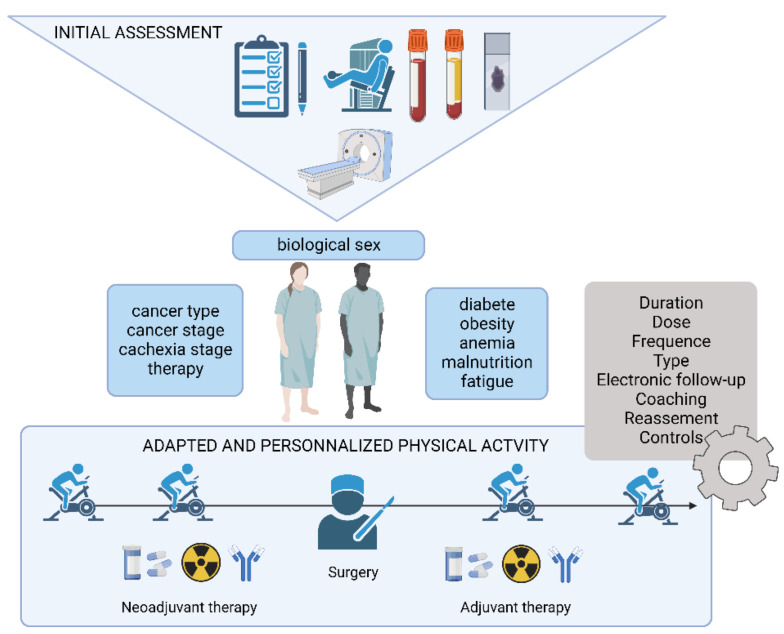
Limits and parameters to include in the APA set-up. The initial assessment must include different markers and observations (e.g., CT scan, questions, physical test, blood draw, biopsies) to help build an APA around the scheme of therapy of the treatment (diagnosis, neoadjuvant therapy, after surgery and after remission). Created with BioRender.com.

However, we need to raise a potential bias. Since most of these studies are randomized controlled trials, they may include patients in better shape and with less associated morbidity, due to the inclusion criteria of the different studies. It seems important that cancer patients seek medical evaluation to inform their physical and nutritional program. This type of advice is invaluable in creating a safe and effective fitness plan for patients with appropriate and tailored modifications, related to a specific cancer diagnosis or treatment-related issues [92,96,97] (Figure 3).

Regardless of the means available, the person should be assessed for inter- and/or intra-individual comparison, in order to propose an adapted management. However, it is important to point out that it is not enough to rely on the final results of the assessments, but to gather as much relevant information as possible that can explain these results and allow for an optimal APA program. For example, the strategy used to stand up from a chair gives more information than the final number achieved over 30 s. Kinematic analysis with a smartphone is within everyone’s reach. As another example, the evolution of the walking speed (every 30 s or every 50 m) during the 6 MWT (6 min walk test, exercise to evaluate the patient function limitations) is more interesting than the total distance covered.

Individualization is essential to optimally improve the qualities required for the patient’s needs. An initial plan can then be produced, based on the assessments and the patient’s APA level and history. The planning, composed of work cycles, with a focus on different physical components, begins with a two-week discovery phase, necessary to seek adherence to the program and to the practice of physical activity. Thus, the playful exercises proposed satisfy a physical and social need that refers to the notion of pleasure with the aim of perpetuating the practice of physical activity following this program. This period is also conducive to taking ownership of one’s body and correctly carrying out the movements requested. In fact, in the first phase of training (approximately one to two weeks), a rapid improvement in the ability to perform an exercise is mainly the result of a learning effect, mediated by changes in the coordination of motor skills.

The notion of the training load, which covers the overall amount of physical activity over a given period of time, combining the parameters of intensity, frequency and duration of the session, is still too often absent from the literature. Indeed, the training load must be gradually increased so that the relative intensity remains high enough to provide an adequate overload throughout the training program. However, the idea is that the training load should not be increased by more than 10% (usually from one week to the next), in order to avoid overtraining and therefore potential ‘bad fatigue’. This 10% guideline, often used in athletes, can be applied to people with cancer but the response of the trained person should be assessed at each intervention by a numeric scale or visual analogic scale. It should be standard practice to assess the rate of effort perception (RPE) after each session and the state of fatigue before each session. This indication allows the instant adaptation and individualization of the content and intensity of the session.

The intensity of the exercises should be moderate (RPE 5-6/10), as increasing the intensity reduces the expected effects on fatigue. The recommended amount of weekly APA with treatments should not exceed 10–12 MET.h/week.

The concept of accumulation is to achieve the goal of 150 min of weekly physical activity by doing several shorter activities—at least 10 min each—spread over a week, and then adding up the time spent on each of these activities, e.g., 30 min of moderate intensity activity five times a week. They can also be applied to older people with disabilities, with adjustments made where necessary to take account of the individual abilities, risks or limitations.

Thus, many questions still need to be addressed to achieve standardization in clinical practice and adapt care to each patient followed for cancer but also to improve the adherence of patients and health professionals to the APA programs.

## 3. Emerging Mechanistic Views

Physical activity and appropriate nutrition are now considered an integral component of “standard of care” therapy in the primary, secondary and tertiary preventions of cancer [99].

Indeed, their impact is important on the factors that control the tumor development, such as the immune system, inflammation, tissue perfusion, hypoxia, insulin resistance, the amount of lactates, metabolism, glucocorticoid levels and cachexia. For more than 20 years, many researchers have studied the effects of physical activity in patients followed for cancer, making it possible to develop a new domain commonly called exercise oncology [100]. A major objective of this emerging research field is to determine the efficacy of, and the biological mechanisms by which, aerobic exercise affects cancer incidence, progression and/or metastasis.

In a systematic review of in vivo preclinical data was published in 2016 [101]. Ashcraft showed that a majority of studies reported that exercise inhibited the tumor initiation or multiplicity, inhibited the tumor proliferation, and decreased the metastatic process. To better understand these results, we must look at the impact of activity and nutrition at different levels: the host, the microenvironment and the tumor.

### 3.1. Systemic Effects

#### 3.1.1. Muscle Secretome during Exercise

**Figure 4:**
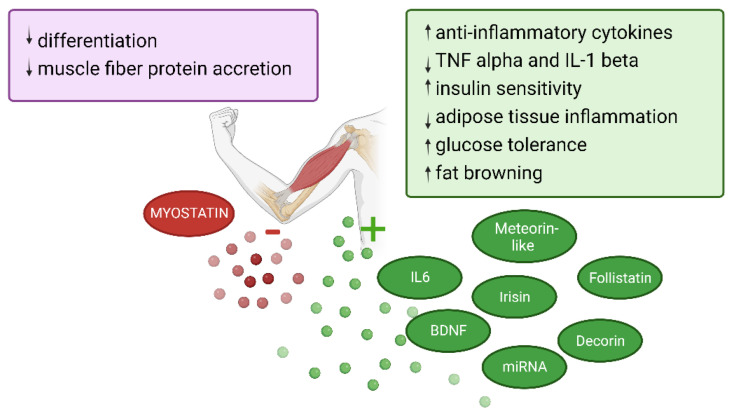
Schematic of the effects of exercise on the muscle secretome and the systemic consequences. Exercise induces an increase in meteorin-like, follistatin, decorin, irisin, BDNF, but also in cytokines, such as IL-6 and miRNAs. This leads to an increase in anti-inflammatory cytokines, in insulin sensitivity, in glucose tolerance and in fat browning, but a downregulation of the TNFα and IL-1β. Myostatin synthesis and secretion is decreased during exercise, causing a decrease in muscle differentiation and muscle fiber protein accretion. The + symbol and the green color emphasize the myokines and miRNA with an increased expression and/or secretion during exercise. The – symbol and the red color highlight the one with a decreased expression and/or secretion during exercise. The dots represent the myokines gradients. Created with BioRender.com.

Similar to adipose tissue, muscle secretes different myokines, which are small cytokines involved in anti-inflammatory, cardiovascular, metabolic and immunological processes [54]. Paracrine, autocrine and endocrine effects of these myokines were detected and described (Figure 4) [102].

The levels and diversity of myokines vary between the type of exercise provided by a person. During and after exercise, the mechanical tension, muscle damage and metabolic stress induce morphological adaptations of the muscle [103] through the protein biosynthesis and myokines secretion. Indeed, once a contraction stimulus is received by the muscle cells, anaerobic metabolites are accumulated and the cells pH level decreases. The hypothalamus will respond to these changes by secreting growth hormones, testosterone, and causing the secretion of anabolic myokines, in particular IL-6. The mTOR pathway involved in the protein synthesis [104] will be stimulated and lead to an increase in the myokine levels. Myofibrils and sarcoplasm will hypertrophy [103].

The first discovered myokine, IL-6 is a cytokine that is primarily pro-inflammatory, but can have anti-inflammatory capacities when released by the muscle [54]. The structural differences between muscle cell IL-6 and other forms of IL-6 are unknown but there are different pathways to activate the IL-6 receptor, inducing proinflammatory or anti-inflammatory effects [105]. Plasma levels of this myokine can increase 100-fold during physical activity [106]. Muscle-derived IL-6 has also a role in the metabolic system, by enhancing the insulin-sensitivity [107].

Myostatin belongs to the transforming growth factor beta family (TGF-ß) [108] and is the only myokine reduced through exercise. The process of agglomeration of the proteins present in the muscle fibers is inhibited by high levels of myostatin [109] and are correlated with sarcopenia [110]. Two studies show a positive link between obesity, high levels of myostatin and insulin resistance [109,111]. Cardiomyocytes can also produce myostatin at high levels, which is correlated with the risk of heart failure [112].

Decorin (myostatin antagonist [113]) overexpression can improve the myoblasts proliferation and supervise the myotubes growing [114].

Follistatin, another important antagonist of myostatin [115], can induce the proliferation of satellite cells, and protein synthesis, leading to muscle hypertrophy [116], but can also accelerate muscle tissue repair after injury in mice and reduce the risk of developing muscle fibrosis [117].

The brain-derived neurotropic factor (BDNF), another myokine, produced by different types of cells (skeletal muscle cells, cardiac myocytes, smooth muscle cells and cells in the liver and brain) [118], has a key role in the endogenous reparation of myocardial and skeletal muscle cells [119,120]. High levels of BDNF activity can reduce insulin resistance, obesity and blood glucose levels [121] via the hypothalamic melatonin pathway. BDNF may protect against obesity by regulating the metabolism [122].

Irisin is one of the most recent myokines described to date [123], and its role is still controversial. Biochemically, it is a PGC-1α dependent molecule. Irisin release seems to be stimulated by resistance training more than endurance training [124,125]. White adipose tissue could turn brown under its action [123], inducing an increase in the expression of the mitochondrial uncoupling protein 1 (UCP 1) responsible for an increase in energy metabolism [126]. Irisin can be produced in muscle, adipose tissue, and in the myocardium [127]. This leads to an improved glucose homeostasis and lipid metabolism, and reduces insulin-resistance and adipose tissue inflammation [126,128].

The last described myokine is meteorin-like and depends on PGC-1α. Like irisin, meteorin-like can induce the transformation of white adipose tissue to brown adipose tissue, via the production of IL-4 and IL-13 expressing eosinophils in the adipose tissue [129]. This leads to an improved glucose tolerance and a higher insulin sensitivity in mice. Moreover, it seems involved in the immune responses and inflammatory regulation [130].

The role of these different myokines, secreted by the muscle in action, revealed the central role of this organ in the regulation of the metabolism and inflammation. However, the stimulation of myokines also seems to depend on various factors, such as nutrition, lifestyle, drugs or circadian rhythm [131]. For instance, a balanced nutrition provides amino acids that can induce the mTOR pathway and lead to protein biosynthesis [132]. Thus, an appropriate physical activity and a healthy diet can then preserve this organ and allow it to function optimally.

A recent review by Papadopetraki et al. [133], summarizes the interesting role of another type of muscle-derived factors induced by exercise, the miRNAs. MiRNAs are single stranded non-coding RNA with an average length of 20 nucleotides and are involved in the post transcriptional regulation of genes.

All of these myokines may influence the crosstalk between organs and the tumor during exercise.

#### 3.1.2. Muscle Crosstalk with Other Organs during Cancer

**Figure 5:**
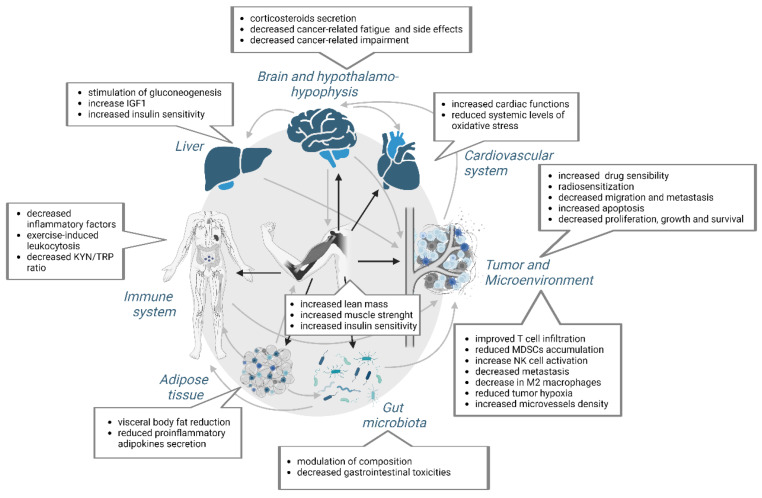
Emerging views of the muscle crosstalk with other organs (brain, hypothalamo-hypophysis, liver, immune system, adipose tissue, gut microbiota, cardiovascular system and tumor and its microenvironment) during the APA leading to mostly, but not exclusively, the anti-tumoral consequences. Created with BioRender.com.

Muscle is intimately linked with other organs in the body, such as the liver, adipose tissue, and the hypothalamus, to regulate the energetic homeostasis [134] (Figure 5). The liver collaborates with the adipose tissue for the metabolism of lipids and glucids. All adaptation mechanisms contribute to maintain an optimal concentration of the ATP in the muscle [135].

TGFβ is an interesting example of a cytokine involved in a crosstalk between organs during cancer. Alterations in the TGF-β signaling pathways has been reviewed in different type of cancers [136,137,138,139] contributing to different aspects of impaired muscle regeneration and sarcopenia [140,141,142,143]. Different elements have been described in human, as well as in mice models, and link cancer and muscle alteration: (i) TGFβ level is found increased in the tumor [144,145] and the serum of patients with different types of cancer [146,147,148], (ii) the TGFβ family has an important role in the regulation of the synthesis/degradation protein balance controlling muscle mass [149] (iii) TGFβ is an important regulator of inflammation and fibrosis [150], two important mechanisms leading to muscle atrophy. Other mechanisms have been described but not necessarily in a cancer context.

Thus, it is essential, when deciphering the effect of exercise during cancer, to consider all of the effects on the different organs.

#### 3.1.3. Effect of Exercise on the Hypothalamo-Hypophyseal-Adrenal Axis

As mentioned earlier, several hormones and signaling molecules are induced during muscular exercise and may play a role in cancer development.

Martin et al. [151] hypothesized that glucocorticoids would be important systemic mediators of cancer cachexia and showed the role of the hypothalamic-pituitary-adrenal-glucocorticoid pathway in the transcriptional regulation of the skeletal muscle catabolism and hepatic metabolism during cancer cachexia [152,153,154,155,156,157]. Cortisol rises during intense, prolonged exercise [158] and stimulates the hepatic gluconeogenesis to counteract the blood sugar levels drop. A potentially negative impact of physical activity has been described through the glucocorticoids secretion. Indeed, the stress-induced activation of the hypothalamus axis and corticosteroids released during exercise can accelerate the tumor growth [60] and have various negative effects on the immune response [159], which counterbalance the potential beneficial effect of exercise on the tumor growth (Figure 5). Similarly, strenuous exercise can increase the IGF1 levels [160] and thus have deleterious effects by promoting the tumor growth. This sort of mechanism may contribute to explain the U-shaped curves of the dose/protection relationship of physical exercise [161].

Therefore, the hypothalamo-hypophyseal-adrenal axis, recently described in the context of tumor-induced cachexia, is an example of the duality of the exercise-induced systemic changes in patients through the IGF1 and corticosteroids release.

#### 3.1.4. Effect of Exercise on Treatment-Associated Toxicities

The APA has been shown to help reduce the acute reactions related to chemotherapy, such as nausea and vomiting (*p* = 0.029 and 0.031, respectively), pain (*p* = 0.003), physical fatigue (*p* < 0.001), and help maintaining muscle strength (*p* = 0.002) [93].

**Figure 6:**
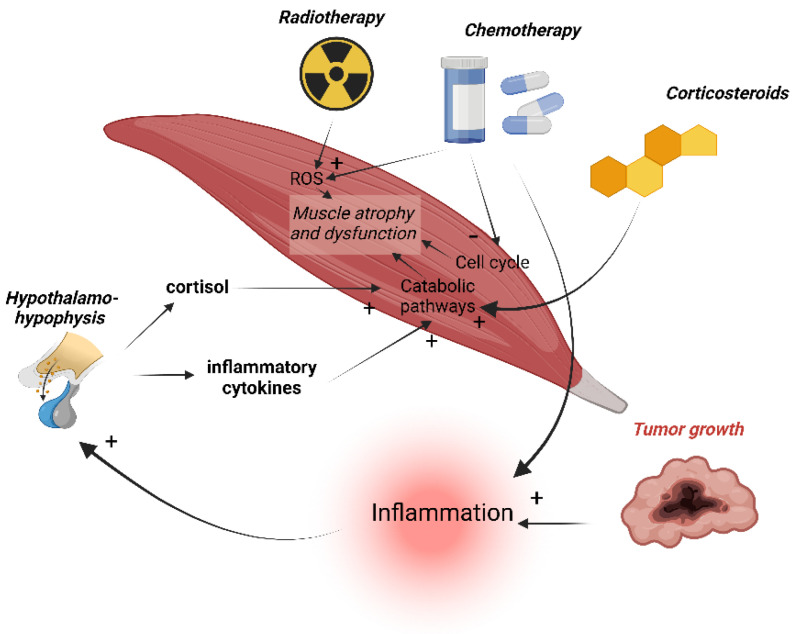
Schematic of the muscle direct- or indirect-related toxicities (muscle atrophy and dysfunction) induced by cancer and anti-cancer treatments (radiotherapy, chemotherapy, corticosteroids). The + symbols represent a stimulation/induction and the – symbols, an inhibition. Created with BioRender.com.

Indeed, chemotherapeutic agents reduce the body mass along with skeletal muscle atrophy and dysfunction [162,163,164,165]. As chemotherapy dosing is based on the body surface area and relies on a lean body mass, muscle atrophy will lead to a decreased dosage to avoid an increased toxicity [166]. Thus, some hydrophobic chemotherapies, such as taxanes, have an increased dose-limiting toxicity when the patient is sarcopenic, confirming the key role of the muscle mass in treatment toxicities [167]. However, irisin produced by the muscle, in response to exercise, enhances the sensibility of breast and pancreatic cancer cells for doxorubicin [168,169] and will reduce this chemotherapy-induced toxicity. Research to date has primarily focused on three chemotherapeutic agents commonly used in clinical practice: doxorubicin, cisplatin and 5-fluorouracil. Campelji et al. [170] proposed a comprehensive review on this topic. Cancer drugs can induce cachectic myopathy through several mechanisms, but some common pathways have been described [162], such as the onset of systemic inflammation after some types of treatments [171] and the consecutive stimulation of the hypothalamic-pituitary-adrenal axis leading to the glucocorticoids secretion and through the activation of a pro-catabolic pathway to muscle atrophy [162,172,173,174] (Figure 6). Importantly, corticosteroids, which are one of the most prescribed drugs in oncology, stimulate the expression of myostatin and lead to iatrogenic muscle loss [175] Additionally, several chemotherapeutic agents have been shown to promote the production of the reactive oxygen species (ROS) in muscle fiber in vitro, leading to an altered myotube morphometry [165,176,177,178]. Chemotherapeutic agents arrest the cell cycle of tumor cells, as well as healthy cells, which proliferate rapidly (except of cardiomyocytes). Thus, satellite muscle cells that rapidly divide and differentiate in response to muscle injury and growth factors are also affected, contributing to the net loss of muscle mass seen in cancer-related cachexia (Figure 6) [179].

Muscle wasting is one of the most relevant negative effects of cisplatin and a major cause for a clinical decline of cancer patients since it is a negative predictor of the treatment outcome and is associated with an increased mortality [163]. Cisplatin-related muscle wasting is correlated with weight loss of up to 30% in in vivo studies [180,181,182,183]. This is caused by an increased catabolism and a reduced appetite. Following the cessation of cisplatin administration, treated animals begin to gain weight again and increase their food consumption, but their body weight remains significantly lower than that of the control animals [180,181]. This muscle wasting leads to muscle weakness and fatigue, mainly related to the depletion of the skeletal muscle mass [184]. Cisplatin-induced muscle dysfunction is caused by the activation of several mechanisms ranging from the impairment of ubiquitin-proteasome, autophagy, and the IGF-1 pathway/PI3K/Akt, to calcium homeostasis and the dysregulation of the lipid metabolism, mitochondrial damage, oxidative stress and the upregulation of pro-inflammatory cytokines [163]. Some treatments have shown their effectiveness in vitro and in vivo, to fight against this effect induced by cisplatin, such as ghrelin [185], growth hormone secretagogues [186,187,188], D-methionine [189] and taurine [190,191] but none is currently used in clinical routine. Exercise can have a major impact on the side effects of chemotherapy drugs, such as cisplatin-induced muscle wasting. Voluntary exercise in mice, during cisplatin treatment, maintained a lean body mass (*p* < 0.001) and muscle strength (*p* < 0.001) [192]. Bae et al. [193] showed that ladder and aerobic exercises in mice directly decrease the cisplatin-related muscle wasting by modulating the AKT/PGC1-α/FOXO3a signaling pathways, regardless of the skeletal muscle type. Exercise could therefore be an easy and inexpensive way to reduce this dreaded cisplatin complication. Moreover, a meta-analysis of the various studies on mice has shown that exercise helps preserve the cardiac function during and after treatment with doxorubicin [194]. Despite this evidence, only a small amount of research has been carried out on therapeutic strategies to protect muscles during cancer treatment.

Cancer-related fatigue (CRF) is a very common and debilitating side effect of chemotherapy and can persist for years after the end of the treatments [195,196]. CRF can also exacerbate others cancer-related side effects, such as depression, anxiety, sleep disturbances and pain [197,198,199]. Mustian et al. [200] showed, in a review of the literature including 113 articles and 11,525 participants, that exercise improved CRF during and after treatment (*p* < 0.001), whereas pharmaceutical interventions did not (*p* = 0.05).

Cancer-related cognitive impairment (CRCI) is a common side effect experienced by numerous cancer survivors and it has a significant impact on their quality of life. Multiple mechanisms are potentially responsible for CRCI and among them, is the direct neurotoxic injury of systemic treatment and radiation [201]. Circulating inflammatory markers are increased in women with breast cancer who underwent adjuvant chemotherapy and are associated with a reduced neurocognitive performance [202,203,204]. Neurocognitive functions after chemotherapy can be improved, by reducing systemic levels of oxidative stress through physical exercise, especially in cancer survivors. In a meta-analysis including 12 studies (936 breast cancer survivors), exercise was found to improve the self-reported cognitive function (*p* < 0.0001), cognitive fatigue (*p* = 0.03) and executive function (*p* = 0.0001) [202]. Moreover, cancer patients receiving chemotherapy and participating in a thrice-weekly exercise program, demonstrated reduced blood 8-hydroxy-2-deoxyguanosine levels (marker of oxidative DNA damage), a significant increase in systemic antioxidant capacity (41%) and a significant decrease in protein oxidation (36%) [205]. Thus, patients who undergo exercise in conjunction with chemotherapy exhibit reduced levels of inflammatory biomarkers and retain their neurocognitive function.

Cytotoxic effects of radiotherapy, used in almost 50% of patient-care [206], are intended to target the transformed cancer cells, but may also have deleterious effects on muscle function [207,208]. Ionizing radiation induces damage to DNA and proteins, leading to cell cycle arrest and cell death [209]. Mitochondrial DNA is also vulnerable to radiation, due to the relatively less efficient damage repair mechanisms [210]. Mitochondrial damage causes an increased production of the reactive oxygen species (ROS), mitophagy leading to the mitochondrial dysfunction, disruption of the ATP production and thus causes a decreased muscle function [211] (Figure 6). The juvenile irradiation of mice significantly disrupted the Ca2+ dependent cell signaling processes and excitation-contraction coupling in the skeletal muscle [208]. On the contrary, exercise promotes the translation of proteins and leads to an increase in the renewal of damaged organelles. The concentration of mitochondria is increased by the positive regulation of PGC1α, a transcriptional co activator involved in the mitochondria biogenesis, and induced by a prolonged exercise [212,213]. To support efficient and repetitive muscle contractions induced by exercise, the Ca2+ machinery must adapt with a more effective Ca2+ handling and signaling [212,213]. A recent study demonstrated that irradiation in mice reduced the force of the fast-twitch extensor digitorum longus muscle by 27%, compared to non-irradiated mice. However, the voluntary wheel running post-irradiation improved the muscle-specific force by 37%, and was associated with a significant increase in the PGC1α expression. This is consistent with an exercise-dependent increase in the mitochondrial biogenesis [214].

Thus, exercise, by maintaining a lean body mass, preserving the cardiac function, reducing oxidative stress and modulating the mitochondria biogenesis is able to mitigate some toxicities induced by cancer treatments.

#### 3.1.5. Effect of Exercise on the Systemic Immunity

Tumors use a variety of mechanisms to diminish the T-cell infiltration and recognition, thereby attempting to evade the immune surveillance and contribute to the decreased anti-tumor immunity, the so-called tumor-editing [215]. Some of the beneficial effects of exercise in patients with cancer have been attributed to the regulation of systemic inflammation (Figure 5).

The Exercise-induced leukocytosis [216], observed in the context of exercise, has been described. This is the mobilization of vascular, pulmonary, hepatic and splenic white blood cells in the peripheral circulation from the first session of physical activity [216]. Thus, a vigorous exercise session of 45 to 60 min increases the concentrations of the NK cells and CD8+ T cells, by approximately 2.5 times [217], but also of the CD4+ T cells [218]. Upon cessation of exercise, the circulating lymphocyte and NK cell counts decline rapidly, even to sub-baseline levels, suggesting that they rapidly return to tissues [219]. The dynamic changes in blood pressure, shear force and epinephrine-mediated stimulation of the beta-2-adrenergic receptors on the surface of the lymphocytes, collaborate to the leukocyte demargination and circulation [220,221]. This intensity-dependent mobilization occurs in proportion to the expression of the beta-2-adrenergic receptors on the lymphocytes, NK cells and CD8+ T cells, responding more strongly than the B cells and CD4+ T cells [217,222,223]. Another study in patients with breast cancer showed that after 12 weeks of supervised exercise, the average number of circulating CD3+, CD4+, CD8+, B or NK cells, was not changed but the percentage of CD4+/CD69+ cells (CD69 is a marker of T-cell activation) increased by 50%, compared to the controls who did not exercise [224]. To our knowledge, the role of exercise on the regulatory T lymphocytes (Treg) has not yet been established and studies diverge on the impact of physical activity on their concentration [225,226]. Another study using MMTV-PyMT mice to study breast cancer, showed the impact of exercise on the lactate plasma (among other metabolites) content, leading to an alteration in the CD8 T cell metabolism and an enhanced antitumoral capacity. This increase in the circulating lactate was also observed in human plasma [227].

Exercise also impacts the humoral immunity. Indeed, B cells are also mobilized by exercise. Short periods of cycling in healthy human subjects increased the circulating levels of several B cell subsets, including immature B cells. However, immature B lymphocytes are very important because they can be redistributed to peripheral tissues for maturation and antigen detection [217]. Exercise also increased the antibody levels after an influenza vaccine, in an elderly population [228].

Kynurenine (KYN), a product of tryptophan (TRP) degradation by the enzymes tryptophan 2,3-dioxygenase (TDO) and indoleamine 2,3-dioxygenase 1 and 2 (IDO1, IDO2), was found increased in multiple cancers and associated with a bad prognosis [229]. Expressed by the tumor cells, immune cells, fibroblast and endothelial cells [230,231], KYN is involved in different mechanisms leading to the tumor immunosuppression (e.g., T cell suppression, T reg differentiation), but also increased the tumor malignancy [232,233,234,235,236,237,238,239,240]. Interestingly, the KYN/TRP ratio was found decreased after exercise in serum from patients with breast cancer (Figure 5) [241].

Emery et al. [242] summarized in a review, the important results for the understanding of exercise-induced immunomodulation as an integral mechanism that prevents cancer growth. In their view, physical activity does not “prevent” the initial stages of cancer development (initiation and initial promotion) but gives greater efficacy against the progression to more advanced stages of cancer. They suggest a greater risk reduction via physical activity for cancers that have a higher tumor mutational load. This positions the immune system in the anticancer effects of physical activity, as an integral mechanism preventing the cancer growth at an early stage. Exercise-induced immunomodulation could explain why the epidemiological evidence shows that physical activity does not prevent de novo neoplasia, but rather reduces the incidence of a more advanced disease. Emery et al. [242] hypothesize that physical activity increases the ability of the T cells to promote the elimination of cancer cells after the onset of the immunogenic mutational events, which in turn facilitates the maintenance of cancer in the equilibrium phase of the immunoediting process, thereby delaying or avoiding the clinical progression and the diagnosis of cancer.

However, it is not known whether these effects influence the tumor immunity, although across the various studies, the modulation of the physiological microenvironment through exercise, seems to improve the anti-tumor immunity.

#### 3.1.6. Effect of Exercise on the Gut Microbiota

The gut microbiome is composed of trillions of organisms present in the gastrointestinal tract. The microbiota plays key roles in the development of the host metabolism, in the regulation of the immune system, and in the host inflammatory responses [243].

It is established that disorders in populations of these organisms have a major role in chronic clinical conditions, notably obesity and diabetes [244], by increasing the production of pro-inflammatory cytokines. The intestinal microbiota has a major role in the development of obesity. It plays a role in the digestion of nutrients, their storage and energy expenditure [245]. Changes in the composition of the microbiota, especially caused by obesity, can disrupt the integrity of the gut, leading to a state of chronic low-grade inflammation [246,247,248]. In fact, the microbiota composition plays a role in the innate immunity, via the toll- and nod-like receptor signaling [249], which in turn is responsible for the low-grade state of inflammation associated with obesity [248]. Physical activity can modulate the composition of the intestinal microbiota and reduce the inflammatory signaling pathways induced by obesity. In a study of 39 obese children, exercise reduced the plasma glucose levels and increased the upper and lower extremity dynamic strength. A metagenomic analysis further revealed a bacterial composition associated with obesity. Exercise altered this profile, greatly reducing the *Proteobacteria* phylum and the *Gammaproteobacteria* class. Additionally, physical activity tended to increase certain genera, such as *Blautia*, *Dialister* and *Roseburia*, leading to a microbiota profile similar to that of healthy children [250]. In this study, the reduction of the NLRP3 inflammasome and the CASP-1 proteins by exercise training, supports the idea that the NLRP3 inflammasome detects danger signals associated with obesity and contributes to obesity-induced inflammation [251].

In addition, the composition of the microbiota has an impact on the development of certain cancers [252,253]. In the case of colorectal cancer, food and gut microbiota have been shown to play a key role in tumorigenesis [254]. Diets play a significant role in different gut microbiota compositions [255], which will produce a number of low molecular weight substrates, including biotin, butyrate, folate and acetate, contributing to epigenetic modulations [256]. Among these intestinal bacteria, *Fusobacterium nucleatum*, whose concentration is directly related to diet, and is associated with colorectal cancer and its prognosis [257].

Hao et al. showed that exercise is able to induce a microbial shift, with an increase in the *Fusobacteria* phylum, and to reduce the circulating inflammatory factors and microbiome metabolites, leading to a decrease in the systemic low-grade inflammation. The phylum *Fusobacteria* may contribute to the beneficial effects of exercise on the reduction of the serological inflammatory factors [258]. In this context, one could hypothesize that exercise would have an indirect effect on tumor growth through the modulation of the microbiota.

Taken together, these data suggest that the microbiota could be one of the intermediate mechanistic links between obesity, inflammation and the cancer risk, and that the APA could exert an antitumor effect by the modulation of the microbiota (Figure 5).

The gut microbiota may also have a direct role in modulating the side effects of cancer treatments. The toxicity induced by radiotherapy on the gastrointestinal tract can lead to the premature discontinuation of the treatment and thus lead to a reduction in the effectiveness of the treatment and the prognosis of patients with cancer. In the United Kingdom, approximately 90% of patients receiving pelvic radiotherapy, have reported alterations in their bowel function, leading to negative effects on daily activity in up to 50% [259]. Wang et al. [226] showed that physical activity protected mice against radiation-induced gastrointestinal tract toxicity, as judged by the denser intestinal villi, more goblet cells, and a lower expression of inflammation-related genes in the small intestine. They highlighted that walking restored the gut microbiota configuration, such as the elevation of *Akkermansia muciniphila*, and reprogrammed the gut metabolome of irradiated mice. Additionally, abdominally irradiated recipient mice that received a fecal microbiome from donors who had physical activity, showed less intestinal toxicity. The oral gavage with *A. muciniphila* attenuated the radiation-induced gastrointestinal tract damage.

In addition, the immune checkpoint inhibitor therapies, such as ipilimumab, predisposes patients to a number of immune-related adverse events, such as colitis, and the ecosystem of the gastrointestinal microbiota may play a significant role in this phenomenon [260]. Interestingly, Cho et al. showed, in mice, that exercise preconditioning alleviated the severity of the clinical symptoms of colitis associated with a high-fat diet (HF) plus dextran sulfate sodium (DSS) treatment, and this appeared to be associated with the symbiotic modifications in the gut microbiota [261].

Thus, the modulation of the gut microbiota through physical activity could offer a therapeutic benefit to radiotherapy- and immunotherapy-associated toxicities.

### 3.2. Tumor and the Microenvironment

The tumor is composed of tumor cells surrounded by the microenvironment composed of ECM, fibroblasts, blood vessels and immune cells, with multifaceted interactions [262].

#### 3.2.1. Local Immunity

As described previously, the interactions between the tumor and the immune system are numerous. The mechanisms behind the antitumor effects of physical training are not fully understood but some in vivo studies attempt to approach the link between the microenvironment and exercise (Figure 5).

The local and systemic effects of exercise contribute to improve the T-cell infiltration in tumors. The circulating levels of IL-6 are increased by exercise. This leads to enhance the adhesion molecules on the tumor vascular endothelium and to promote the traffic of T-cells [263,264]. Further, exercise modifies the distribution of the NK cells which have a cytotoxic activity against the cancer cells in vitro [265,266].

The tumor progression is associated with the marked splenic accumulation of immunosuppressive myeloid-derived suppressor cells (MDSCs) with a protumor action [267]. In a mouse model of triple-negative breast cancer, exercise slowed the tumor progression and reduced the tumor-induced accumulation of MDSCs [268]. In mice bearing 4T1 tumors, a reduction in MDSCs was reported and was proportional to the level of physical activity [269]. The MDSC reduction was accompanied by a relative increase in the NK and CD8 T cells activation. Pedersen et al. [266] showed that exercise, prior to melanoma implantation in mice, exhibited a 6-fold increase in the NK cell infiltration into the primary tumors, reduced the tumor growth rate by 60% and halved the number of lung metastases, compared with the sedentary controls. An increase in the NK cell activation has also been reported in mice and patients who exercise [266,270] and could contribute to the observed trend of a reduced metastatic burden as the NK cells are involved in the control of the micrometastatic disease [270,271]

Another in vivo study of mice with established breast cancer showed that the CD8+ T cells were responsible for the antitumor effect of exercise. The recruitment of the CD8+ T cells and the beneficial effects of exercise were abrogated in the Cxcr3−/− mice, confirming the causal role of the CXCL9/CXCL11-CXCR3 pathway. Thus, physical activity was able here to reprogram the microenvironment immune system, and to enhance the antitumor activity mediated by the CD8+ T cells via CXCR3 [272]. Wenneberg et al. [268] found that exercise improved the tumor response to focal radiotherapy and the programmed cell death (PD-1) blockade, suggesting that it may be a component of a multimodal breast cancer treatment that includes immunotherapy [273].

A recent study on pancreatic cancer confirmed the exercise induced a decrease in MDSCs and the shift in the T lymphocytes toward the effector and cytotoxic phenotypes in mice. They also demonstrated that aerobic exercise sensitized the pancreatic tumors to the α-PD-1 therapy through the IL-15Rα+ CD8 T cells in mice. Finally, an exercise-dependent increase of the intra-tumoral CD8 T cells was also observed in humans [274].

The tumor-associated macrophages (TAM) also contribute to the innate antitumor immunity by secreting proinflammatory cytokines (e.g., IFNγ and IL-12), which support the NK cell activation (M1 macrophages) [275]. In advanced cancer, TAMs differentiate to a protumoral state (M2 macrophages) and secrete immunosuppressive cytokines, such as IL-10. Exercise could avoid polarizing the macrophages to the M2 phenotype in the tumor and promote the antitumor/M1 polarization of the peritoneal macrophages [276].

These studies decipher the different mechanisms leading to the immune-mediated tumor regression induced by exercise, the T cells activation seems to be one of the most conserved mechanisms between these studies.

#### 3.2.2. Fibroblasts and the ECM

Upon the development of cancer, the tumor will rewire the surrounding fibroblasts and mesenchymal cells to promote fibrosis locally, but also systemically in different organs, during the development of the cachexia syndrome (muscle or adipose tissue). The infiltration of the immune cells into the fibrotic tissue plays a key role in amplifying the fibrotic response, by secreting several cytokines and chemokines responsible for the differentiation of the myofibroblasts [277]. TGF-β is a cytokine essential for the induction of the fibrotic response and the activation of cancerous stroma, by the development of carcinoma-associated fibroblasts (CAF), which promote the progression of the disease by providing cancer cells with proliferative, migratory, survival and invasive signals [278]. In addition, TGF-β exerts a pro-tumoral activity by inhibiting the host tumor immunosurveillance. In a study where patients undergoing chemotherapy and/or radiotherapy treatment followed a fitness program on Xbox^®^, a significant reduction in the proinflammatory cytokines was observed (IL-6: *p*< 0.05; IL-10: *p* = 0.038; TGF-β: *p* = 0.049) [279]. In another study, the levels of TGF-β1 in the recreational cyclist group were lower than the sedentary group, showing that regular physical exercise triggers exercise adaptations that can suppress the latent TGF-β1 activation [280].

Therefore, more studies are need to be carried out to confirm this effect of exercise on the TGFβ level and to decipher the underlying mechanisms.

#### 3.2.3. Angiogenesis

The majority of tumors exhibit a tortuous vasculature, characterized by shunts, a low microvessel density, and a low pericyte coverage [281]. An aberrant tumor vasculature results in pockets of hypoxia, invasion, metastasis [282] and the downregulation of the leukocyte entry into the tumors [215,283]. Tumor angiogenesis and vascular normalization to increase oxygen and drug delivery, are targets of anti-tumor therapeutics [284]. Several reports have shown conflicting results on the effect of exercise on tumor VEGF levels [285]. Indeed, VEGF was decreased in breast cancer survivors with a weight loss intervention but did not change in those participating in an exercise only trial [286].

Hypoxia is present in most of the solid cancers and contributes to the treatment resistance and decreased life expectancy in cancer patients [101,287,288]. Hypoxia inhibit the macrophage and the NK cell activities [289], and the adaptive function of the immune cells by disrupting the balance between the effector T cells and Tregs [290]. Several studies show that exercise reduces the tumor hypoxia [290,291]. In rats, exercise doubled the tumor blood flow, thereby increasing the O2 delivery to the tumors. The hypoxic fraction of the tumor was reduced by up to 15% [292]. The microvessel density is also increased during physical activity, reducing the hypoxic tumor fraction, compared to sedentary controls [293,294,295]. The observed differences in the microvessel density and perfusion may reflect how exercise reduces the oxidative stress [293]. The mechanisms underlying the improvement in the tumor growth retardation with the combination of exercise and chemotherapy, are many. The increase in the functional tumor microvessels (the concept of vascular normalization) increases the chemotherapy exposure to a greater portion of the tumor cells, by eliminating the shunts that leave certain regions unexposed to the drug. Alvarez Florez Bedoya et al. [296,297] showed that moderate exercise reshapes the tumor vasculature and increases the efficacy of gemcitabine against the patient-derived xenograft pancreatic tumors. Exercise, by reducing tumor hypoxia, also makes it possible to increase the efficacy of immunotherapy because hypoxia modifies the infiltration and inhibition of various immune cells [100].

The treatment response depends on the well-oxygenated tissue. Radiosensitivity is reduced when partial pressures of oxygen are lower. Exercise can influence the tumoral vasculature and oxygenated blood perfusion, thereby reducing hypoxia. Exercise could act, in this way, as a radiosensitizer and improve the efficacy of the immune therapy [292,294,295,298] (Figure 5).

Exercise seems to be an important modulator of the tumor vasculature and hypoxia, leading to the potentiated response to different types of therapy.

#### 3.2.4. Effect on the Tumor Cells

Physical activity also has an impact on the tumor cell itself [101]. Brown et al. [299] proposed an important mediating role of physical activity through the redox signaling. Alterations in the skeletal muscle reactive oxygen species (ROS) levels can alter the muscular secretome and, in particular, the secretion of myokines which can modulate the systemic inflammation. Differing levels of the ROS can also modulate the metabolism, hormones and angiogenesis [300], resulting in tumor radiosensitization. It can also potentially reduce the expression of the prometastatic genes [294,297,301,302].

Moreover, exercise has a direct effect on the signaling pathways involved in tumorigenesis. In a mouse model of liver tumors (Alb-Cre; Pten^flox/flox^), the number and size of the liver tumors were reduced through exercise [303]. Mechanistically, exercise stimulated the phosphorylation of AMPK and its substrate raptor, which decreased the activity of a central regulator of the cell proliferation, growth and survival [304], the mTOR kinase in the tumor cell [305].

Most results suggest the antitumoral effects of irisin [306], by inducing cancer cell apoptosis and reducing the migration of cancer cells [169]. Lower levels of irisin have been highlighted in breast cancer patients, compared to a healthy population [307]. The antitumoral effects of irisin have been reported in preclinical studies with colorectal, prostate, lung, bone and pancreatic cancers [308,309,310].

Another interesting mechanism has been described in the triple-negative breast cancer mouse model [311]. In this study, they showed that moderate aerobic physical intervention after the graft of the tumor, had a negative impact on the tumor growth through a modulation in the tumor cell metabolism. Indeed, they observed a decrease in the mitochondrial activity (essential for tumor growth) due to a decrease in the respiratory chain capacity. In this study, they also highlight the fact that the tumor subtype, the number of inoculated tumor cells and the characteristic of the exercise can modulate the anti-tumoral effect of exercise.

Within the crosstalk between the muscle and the tumor microenvironment, decorin, which is secreted during exercise, has been shown to inhibit angiogenesis, carcinogenesis but also the TGFβ-induced fibrosis, through the interactions with different receptors, such as the epidermal growth factor receptor (EGFR) or insulin-like growth factor 1 receptor (IGF-1R), regulation of cell-cycle associated genes, but also miRNAs [312,313,314]. Moreover, its high level is associated with the improved physical function and an increased overall survival (median 732 days vs. 463 days) in hepatocellular carcinoma [315].

MiRNA secreted by the muscle, in response to exercise have also been involved in the direct tumor growth regulation through the exosome-mediated tumor cell delivery. One of them, miR-206, is directly produced by the muscle, and has a tumor suppressor effect through a reduced cell invasion, increased apoptosis and reduced cell growth [133].

Finally, the Hippo signaling pathway has been described as deregulated in multiple cancer types, leading to a cellular level to cell cycle deregulation, epithelio-mesenchymal transition but also to immunogenicity and various therapy resistances [316]. In breast cancer, the activation of the YAP/TAZ oncoproteins is associated with a poor prognosis [317], and in gastric cancer and hepatocellular carcinoma, the YAP-targeting molecules cause the pronounced tumor regression in mice [318,319,320,321,322,323,324]. Endurance exercise has been suggested to alter the Hippo signaling pathway in the skeletal muscle through AMPK, catecholamine, and the IL-6-driven YAP regulations [325,326]. A major effect of an activated Hippo signaling, and thus a reduced YAP activation, is a pronounced suppression of the tumor formation (Figure 5) [317,318,319,327,328]. Tumorigenesis was shown reduced by 50%, when the cancer cells were preincubated with serum after exercise before being injected into the mice. This effect was blunted by the blockade of the β-adrenergic signaling, indicating that the catecholamines were responsible for this tumor reduction [326] An increased catecholamines release, during exercise, also leads to cardiac activity stimulation and an increase in the blood pressure, among other effects.

Although multiple studies performed on mice allow to make direct links between exercise and diverse anti-tumoral effects, these links are usually missing in human studies (Figure 5). Moreover, long-term studies in patients would help to better understand the long-term benefits of physical activity in cancer patients.

## 4. New Applications

### 4.1. Future of the Clinical Research

Despite all of its described potential beneficial effects, it remains challenging to conclude on the real efficacy of exercise and to increment it into the everyday cancer care practice. Indeed, several parameters negatively affect the statistical significance of the observed clinical and preclinical results and prevent us from being able to establish the overall recommendations. These limits are related to the heterogeneity of the patients and their general health condition, the disease, the treatments, but also the physical exercise program and the methods of the study itself (Figure 3). One of the big caveats in clinical trials is the weaknesses of the control arms to assess the clinical efficacy of the exercise in patients: (i) it is difficult and almost impossible to do blinded studies, and randomization against no intervention is challenging ethically, and to maintain patient willingness in participating in the study, (ii) there is a risk of “contamination” of the control group without intervention (i.e., patients are made aware of the interest of the APA and may practice more exercise than the intervention group) and (iii) it is hard to monitor the effective dose of the APA as it includes, in addition to the supervised exercise, the daily activities of the patient. However, these controls are essential to study the additive effect of a structured intervention versus standard care. In addition, the definition of exercise practice (type, dose, timing) varies between studies and international guidelines are lacking to homogenize the procedures between studies. Finally, the APA program should be really adapted to the patients. Indeed, different types of cancer imply varieties of exercise adapted to the debilitating symptoms associated to each cancer (e.g., cardiopulmonary functions of a lung cancer patient, integrity of the bones in a bone metastatic patient, muscle function of a pancreatic cancer patient). Other conditions and their particularities should also be considered as obesity or diabetes.

The classification of the cachexia stages is essential for diagnosing and treating cachexia and should be used for the patient stratification, as we do not know if interventions when cachexia is established will have any significant effect, i.e., if we should increase the intensity or if this is pointless [329]. Similarly, data about the effect of the APA in the context of frailty (another emerging syndrome in oncology overlapping with malnutrition, sarcopenia, and cachexia), are lacking. Complementary examinations could be carried out in clinical practice, in order to objectify the physical condition and the baseline biological status of the patient (Figure 3): (i) a serological test for the different cytokines and metabolites would help define the initial metabolic imbalance and inflammatory state [330], as there may be changes (CyclinD1, Pax7, MyoD and Myogenin) long before the detectable weight loss [331] (ii) assess the muscle mass by CT scan, particularly in obese patients where fat can sometimes mask a loss of muscle mass, could make it possible to prevent certain side effects or to adapt the doses of Prado CM systemic treatments. Body composition in chemotherapy: the promising role of CT [332], (iii) a metabolic assessment using a calorimeter to classify patients [333], (iv) a biopsy could be performed upon the inclusion in clinical trials to develop validated, tangible and reproducible endpoints [334].

It is difficult to know exactly what physical activity is performed in the control groups and the exact “dose” of physical activity received in the intervention groups. One option would be to develop a reliable and reproducible way of monitoring the patients included in the control groups, such as the implementation of connected bracelets (e.g., Garmin^®^ physical activity tracker) or regular detailed questionnaires (e.g., GPAQ, IPAQ). Outside of the trials, wearing a physical activity tracker and/or a pedometer could increase physical activity and improve the health-related outcomes in the individuals with cancer [335]. The systematic literature shows that tracker-based interventions increase physical activity alone or combined [336]. Indeed, wearing a tracker is considered an intervention and an increased motivation by information and peer mentoring [337].

Physical activity must be adapted to the time and type of treatment because its objectives are vastly different (Figure 3). Prior to surgery, the main objective, in combination with nutrition and psychosocial interventions, is to increase the cardiopulmonary function to reduce post-operative complications, in so called prehabilitation programs [338]. The APA can also potentiate the action of immunotherapy with its role on inflammation. Indeed, exercise promotes the survival and proliferation of naive T cells, the generation and persistence of memory cells after exposure to antigens. Thus, exercise improves the efficacy of T cell therapies by improving the quantity, diversity and function of the T cell subsets in cancer patients [339]. Different preclinical studies have tested immunotherapy in combination with exercise on lung carcinoma, breast cancer and melanoma, showing the effect of exercise on checkpoint inhibitor expression and sensitization to the immune checkpoint blockade [272,340,341]. A study in humans assessed the feasibility of the multimodal supportive care program to the metastatic melanoma patients being treated with pembrolizumab [342], but ERICA (Exercise inteRaction Immunotherapy Chemotherapy and cAncer) study currently underway is the first study to assess the feasibility and effects of acute physical exercise carried out in the hour preceding an infusion of immunotherapy (pembrolizumab) and chemotherapy (doublet, based on platinum) in patients with non-small cell lung cancer metastases [343]. In addition, during the adjuvant treatment, exercise potentiates the effect of the treatment by allowing higher doses of treatment, while reducing the side effects associated with the treatments [344,345]. Following radiotherapy, the APA increases the muscle function and reduces the risk of radiation fibrosis syndrome in muscle. Indeed, the prefibrotic phase, constitutive organized phase and the late fibroatrophic phase will progress indefinitely, starting only a few weeks after radiation or sometimes, years after [135,346]. Therefore, it is imperative to target the right moment but also the right dose for the realization of physical activity.

Some studies have tested the combination of appropriate physical activity with systemic treatments but none of these therapeutic strategies is validated today. Some treatments are suggested in the ASCO and ESMO guidelines with a low level of evidence, such as short courses of corticosteroids which aim to increase the appetite, progestins, olanzapine, but these treatments present limiting side effects [2,5]. Anti-inflammatory molecules injections [347,348,349,350], creatine supplementation [351,352] or erythropoietin (EPO) administration to fight against anemia [353] are under evaluation. Of note, to maximize the probability for pharmacological interventions to show an effect, they should be integrated within multidisciplinary interventions (i.e., not to repeat failures, such as with the ghrelin receptor agonist anamorelin), in combination with nutrition and the APA, and take into account the patient heterogeneity (e.g., stratification on the cachexia stage or metabolic status).

### 4.2. Future of the Preclinical Research

Protocols used in the preclinical studies should also be adapted to what is completed and can be accomplished in humans. First, the schedule chosen to start the procedure should mimic what is observed in the human clinic. Indeed, most of the patients are already greatly invalidated by the cancer cachexia at diagnosis. Thus, the APA needs to be adapted to their condition, which is not what is being performed in mouse model where most of the models are not metastatic and only at a pre-cachexic state. Furthermore, voluntary activity should be standardized between mice to be comparable. For that purpose, exercise could be followed and measured (a detector in the wheel, for instance) so it can be stopped (by blocking the wheel) when it reaches a defined level of intensity or duration. This structured exercise will resolve the apparent antagonism between the voluntary exercise and exercise training [101]. 

Preclinical models remain great tools to perform longitudinal analyzes that cannot be carried out in humans and offer great opportunities for the deep understanding of the molecular process using different materials, such as muscle biopsies, but also genetically modified models.

Finally, in cancer-associated cachexia research, a focus on less studied organs, and mechanisms, such as bone, the peripheric nerval system and the direct metastasis effect, would help to have a more global and comprehensive approach to cachexia, and thus, a personalized APA.

## 5. Conclusions

The benefits of physical activity seem multiple in the management of patients with cancer, whatever the stage. Physical activity plays a leading role in the prevention (primary, secondary and tertiary) of cancer. However, many studies show a benefit of physical activity on the HRQoL without an impact on the survival. Methodological biases could partially account for this lack of power and are a path for improvement. This may also suggest the appearance of a balance between the muscle and cancer effect: even if the clinical benefit of exercise is real, it may be offset by the cancer signals when the disease or cachexia is too advanced. This also raises the question of whether cachexia and frailty may also affect the adherence to the APA.

Many questions remain unanswered today. The field of research, concerning physical activity and nutrition, continues to expand as our knowledge improves. We have tried, through this review, to obtain a global approach to this area of expertise while giving avenues for potential future research. Moving toward a more personalized, integrated, patient-centered, multidisciplinary management, by trying to understand the different interactions between the cancer and the host as well as the impact of the disease and the treatments on the different organs, seems the most promising to improve the care of cancer patients.

## Figures and Tables

**Table 1:** Definitions of the terms used to characterize exercise in the clinical setting.

Term	Definition
Adapted physical activity (APA)	The exercise intervention structured and supervised by a professional must be individualized for each patient, according to their preferences, disease, treatments, symptoms, in order to be feasible and safe for the patient.
Resistance training	Exercises training muscles against an external force, usually shorter than endurance training.
Endurance training	Repeated isotonic exercises that last in time to improve aerobic capacity [10].
MET	Metabolic equivalent used to quantify the induced energy expenditure during exercise, means of a standardized program derived from the Compendium of Physical Activities, unit: minutes/week [10].
Light physical activity	Less than 3 METs: activities resulting in little or no increase in breathing or heart rate
Vigorous physical activity	More than 6 METs: moderate to large increases in breathing and heart rate [10].
Sedentary behavior	“Any waking behavior characterized by an energy expenditure less than or equal to 1.5 METs, while in a sitting, reclining or lying posture” [11].

**Table 2:** Recent controlled studies about the safety and efficacy of physical activity interventions in advanced cancers.

Author	Cancer’s Type	Age	Number of Patients in Exercise Group	Number of Patients in Control Group	Description of Intervention	Significant Improvement with Exercise	Non-Significant Improvement with Exercise	Dropout Rate
Cheville et al., 2013 [70]	Lung and colorectal cancer; Stage IV	63.8 ± 12.5	33	33	Eight-wk homebased resistance exercise and walking exercise	Ambulatory Post-Acute Care Daily Mobility Short Form (*p* = 0.02) Fatigue (*p* = 0.03) Sleep (*p* = 0.05)	Ambulatory Post-Acute Care Daily Activities Short Form HRQoL	15%
Cormie et al., 2013 [71]	Prostate cancer; secondary bone metastases	73.1 ± 7.5	10	10	Twelve-wk sup low-level aerobic exercise and resistance exercise, targeting major muscle groups	Physical function (*p* = 0.016) 400-m Walk (*p* = 0.010) Body lean mass (*p* = 0.026) Lean mass (*p* = 0.003)	Fatigue HRQoL	25%
Galvao et al., 2018 [72]	Prostate cancer; bone metastases	69.7 ± 7.6	28	29	Twelve-wk sup, combined aerobic exercise, resistance exercis, and flexibility exercise	Physical Function (*p* = 0.03) Leg Extension (*p* = 0.03)	Four hundred m Walk Test Up and Go Test Lean Mass Body Fat Mass Fatigue	14%
Henke et al., 2013 [73]	Lung cancer Stage IIIA/IIIB/IV	NA	25	21	Three chemotherapy cycles long Combined aerobic exercise and resistance exercise	Staircase Walking (*p* = 0.05) Physical Functioning (*p* = 0.02) Cognitive Functioning (*p* = 0.05)	HRQoL Emotional Functioning Symptom	34%
Neuzillet et al., 2022 [83]	Advanced pancreatic cancer	64	157	156	Sixteen-week APA program	Global Health Status, Physical Functioning, Cognitive Functioning, Social Functioning, Appetite Loss	Insomnia, Financial Difficulties, Constipation	17%
Oldervoll et al., 2011 [74]	Incurable, metastatic cancer and life expectancy of 3–24 months: gastrointestinal, breast, lung, urological	62.6 ± 11.3	121	110	Eight-wk sup, combined exercise and aerobic exercise	Shuttle Walk test (*p* = 0.008) Handgrip Strength (*p* = 0.01)	Total Fatigue Physical Fatigue Mental Fatigue Sit-to-Stand	29.4%
Pyszora et al., 2017 [75]	Advanced cancer patients, admitted to palliative care: urogenital, lung, hematological, digestive cancer	72.4 ± 9.5	30	30	Two-wk physiotherapeutic exercise	Fatigue Severity (*p* < 0.01)	Depression Anxiety	NA
Rief et al., 2014 [76]	Cancer patients with metastatic progress: lung, prostate, breast, renal, melanoma	61.3 ± 10.1	30	30	Two-wk sup isometric resistance exercise followed by 12-wk home-based training	Thirty s Sit-to-Stand (*p* < 0.001), HRQoL (*p* = 0.01) Fatigue (*p* = 0.01) Pain (*p* = 0.003)	Functional Interference Emotional Interference Cognitive Interference Overall Survival Progression-Free Survival	20%
Tsianakas et al., 2017 [78]	Recurrent advancing or metastatic cancer: prostate, gynecological, hematologycal, breast, colorectal	65 ± 11.7	21	21	Twelve-wk walking intervention	none	HRQoL Global Fatigue Score	35%
Zhou et al., 2017 [77]	Advanced nasopharyngeal cancer stage III/IV	NA	57	57	Tai Chi exercise (24-form Yang style) 5 h per week	Fatigue (*p* < 0.05) General Fatigue (*p* < 0.05) Physical Fatigue (*p* < 0.05) Emotional Fatigue (*p* < 0.05)	Mental Fatigue	27%
Zimmer et al., 2018 [79]	Metastasized colorectal cancer	68.5	17	13	Eight-wk sup exercise, combining endurance, resistance exercise, and balance exercise	Muscle Strength (*p* = 0.002)	Physical Well-Being Functional Well-Being Social Well-Being Emotional Well-Being HRQoL	20%
Buss et al., 2009 [80]	Advanced cancer patients; short lifetime expectancy	NA	38	19	Four-wk sup, individualized kinesiotherapy	Fatigue (*p* < 0.001) Diminution Intensification Physical Symptoms (*p* < 0.05)	QoL	24.5%
Zhao et al., 2015 [81]	Head and neck squamous cell cancer, Stage III and IV	57 ± 7	11	9	Fourteen-wk resistance exercise and walking exercise	Vitality/Fatigue (*p* < 0.05) Mental Well-Being (*p* < 0.05) Strength Knee Extension (*p* < 0.05) Mental Well-Being (*p* < 0.05)	HRQoL BMI Lean Body Mass Physical Activity	15%
Zhou et al., 2017 [82]	Ovarian cancer stage III and IV	57.3	74	70	One hundred and fifty minutes per week of moderate-intensity exercise	Attention Control (*p* = 0.02), Social (*p* = 0.02), General Health (*p* = 0.004)	Physical Functioning	13.4%

HRQoL: Health-related quality of life.

**Table 3:** Recapitulating the adapted physical activity principle and the tools to facilitate the application.

APA Characteristics	Specificity to Include	Tools
Progressive	Tolerance	A facilitator, regular assessments
	No harmful effects	phase of learning, defining a training load
Regular	Autonomy	Supervision
	Commitment	motivational interview, playful exercises
Patient-specific	Type of cancer	medical history
	Stage of the disease	initial assessment
	Treatment	medical history
	Side effects of the treatment	initial assessment
